# Antioxidant and Antisteatotic Activities of Fucoidan Fractions from Marine and Terrestrial Sources

**DOI:** 10.3390/molecules26154467

**Published:** 2021-07-24

**Authors:** Zeinab El Rashed, Giulio Lupidi, Elena Grasselli, Laura Canesi, Hala Khalifeh, Ilaria Demori

**Affiliations:** 1 Department of Earth, Environmental and Life Sciences (DISTAV), University of Genoa, 16132 Genoa, Italy; Zeinab.ALRashed94@hotmail.com (Z.E.R.); elena.grasselli@unige.it (E.G.); laura.canesi@unige.it (L.C.); 2 Rammal Rammal Laboratory (ATAC Group), Faculty of Sciences I, Lebanese University, Beirut 1003, Lebanon; hala-khalifeh@hotmail.com; 3 School of Pharmacy, University of Camerino, 62032 Camerino, Italy; giulio.lupidi@unicam.it

**Keywords:** *Cystoseira compressa*, *Eucalyptus globulus*, fucoidan, antioxidant, antisteatotic, NAFLD

## Abstract

Fucoidan is a fucose-rich sulfated polysaccharide typically found in the cell wall of marine algae but also recently isolated from terrestrial sources. Due to a variety of biological activities, including antioxidant properties, fucoidan exhibits an attractive therapeutic potential against a wide array of metabolic diseases associated with oxidative stress. We used FTIR, ^1^H NMR and ^13^C NMR spectroscopy to investigate the structural features of a fucoidan fraction extracted from the brown alga *Cystoseira compressa* (CYS). The antioxidant potential of CYS was measured by DPPH, ABTS and FRAP assays, which revealed a radical scavenging capacity that was confirmed in in vitro cellular models of hepatic and endothelial cells. The same antioxidant effects were observed for another fucoidan fraction previously identified in the terrestrial tree *Eucalyptus globulus* (EUC). Moreover, in hepatic cells, CYS and EUC exhibited a significant antisteatotic action, being able to reduce intracellular triglyceride content through the regulation of key genes of hepatic lipid metabolism. EUC exerted stronger antioxidant and antisteatotic effects as compared to CYS, suggesting that both marine and terrestrial sources should be considered for fucoidan extraction and therapeutic applications.

## 1. Introduction

Plant and algal cell walls are dynamic and complex structures rich in polysaccharides, which gained research attention due to their high potential in fiber, food, nutraceutical and pharmaceutical industries [1]. Recently, we reviewed the effects of brown-algae polysaccharides as active compounds against nonalcoholic fatty liver disease (NAFLD) [2], a global burden affecting 25% of the population worldwide, whose worsening is closely associated with liver failure, insulin resistance and cardiovascular diseases [3].

First identified by Kylin in 1913, [4] fucoidan (FU) is one of the most abundant brown-algae polysaccharides. It is characterized by an high abundance of sulfate groups bound to a backbone of α-(1–3)-linked fucose units or α-(1–3) and α-(1–4) alternating linked fucose residues [5]. FU attracted great interest in research due to its therapeutic potential as an antioxidant, anti-inflammatory, antiviral, antitumor and antisteatotic agent [6]. Interestingly, FU has also been found in marine invertebrates such as sea urchin and sea cucumber [7] and in land plants largely used in Lebanese traditional medicine, such as *Ferula hermonis* [8] and *Eucalyptus globulus* [9].

In this work, we extracted a FU fraction from the brown alga *Cystoseira compressa* (Esper) Gerloff and Nizamuddin (CYS) harvested on Lebanese coasts. We discussed chemical and structural characteristics of CYS and we studied its antioxidant and antisteatotic properties using in vitro assays and experimental cellular models related to oxidative stress-related metabolic diseases such as NAFLD and atherosclerosis. We used rat hepatoma FaO cells and human endothelial HECV cells exposed to a fatty acid mixture: the former are a reliable in vitro model for NAFLD [10], and the latter represent an in vitro model of vascular endothelial damage associated with NAFLD [8,11]. In these models, we also tested the antioxidant and antisteatotic abilities of a FU fraction from *E. globulus* Labill (EUC), previously extracted and characterized as mentioned above [9].

## 2. Results and Discussion

### 2.1. Extraction Yield and Chemical Composition

The marine environment is possibly an underestimated source of bioactive compounds that could be considered in view of the purification of nutraceuticals and phytochemicals against a wide range of diseases. Fucoidan (FU) is one of the main brown-algae polysaccharides and is being extensively studied for its pharmaceutic applications [6,12]. We extracted a FU fraction from the brown alga *C. compressa* (CYS), obtaining a yield of 3.6%. Even if a yield of 6.44% has been reported for *Cystoseira barbata* [13], our result is similar to those obtained from other species of the same genus, such as *C. crinita* (2.8%) and *C. sedoides* (3.3%), as well as that previously obtained for *C. compressa* (3.7%) [14]. The yield was also comparable to those from land plants, such as *E. globulus* (EUC, 2.1%) [9] and *F. hermonis* (3.07%) [8]. The presence of sulfated polysaccharides in seaweeds is not surprising, since it facilitates water retention in extracellular matrices, thus being useful for marine algae adaptation to a high salt environment and to prevent desiccation in low tide conditions [15]. Similarly, in land plants, FU could be related to resistance to drought, and its abundance, chemical composition and structural features could be diverse depending on the season, climate conditions, soil composition, etc.

The chemical compositions of CYS and EUC, reported in Table 1, revealed a complex structure with different traces of monosaccharides. The different colorimetric assays confirmed that CYS and EUC were composed primarily of fucose, with a high content of glucose and lower amounts of other monosaccharides, such as galactose and mannose. The molar ratios indicated that the monosaccharide composition was equal in both extracts, similarly to the molar ratio of fucose with respect to sulfate, which was 1:0.1 in both cases. The low protein contamination (about 0.5%) indicated a high level of purity of both extracts. In fact, a protein content ranging from 0.2% to 2.5% has been reported in FU extracted from different sources [16,17,18,19]. Given that natural polysaccharides can be associated with a certain amount of phenolic compounds [20], we also measured the total phenolic content (TPC) and discovered that it was very much higher in EUC than in CYS. However, it is known that the results of TPC measurements may be overestimated due to the contribution of nonphenolic reducing agents, such as sugars and amino acids, reacting with the Folin–Ciocalteu reagent [21]. However, regardless the nature of the reducing components, this result allows hypothesizing a higher antioxidant ability for EUC as compared to CYS.

### 2.2. Spectroscopic Characterization of CYS

A preliminary analysis of CYS structure was conducted by FTIR and proton (^1^H) and carbon (^13^C) NMR. Here, we describe major information obtained from the FTIR spectrum, which is reported in Supplementary Materials (Figure S1), together with the NMR results (Figures S2 and S3) and a general discussion. We did not perform the same analyses on EUC because the extract we used had been previously characterized by the same methods [22].

As shown in Figure S1, the FTIR spectrum of CYS showed a wide band at 3337 cm^−1^, which was assigned to hydrogen-bonded O-H stretching vibration. A weak signal at wavelength 2926 cm^−1^ was assigned to C-H stretching of the pyranose ring [23]. Furthermore, a small peak at 1723 cm^−1^ was due to C=O bond of carboxylate group. The band at 1643 cm^−1^ contributed to asymmetric vibrations of carboxylate anions O-C-O of uronic acid and derivatives. The bands at 1125 and 1027 cm^−1^ were assigned to S=O stretching vibration of the sulfate group and sulfate ester groups, respectively [9]. The band at 866 cm^−1^ corresponded to sulfate peak [24]. Moreover, the band at 580 cm^−1^ represented the contribution of the C-C-H stretching vibration [25,26]. Altogether, FTIR and NMR spectra of CYS indicated the presence of main functional groups that characterize FU.

### 2.3. Radical Scavenging Capacity

Differences in chemical composition, structural variations and molecular weight can affect the bioactivity of purified polysaccharide [5]. The hydroxyl hydrogen donor groups detected by FTIR spectroscopy as well as TPC could contribute to the antioxidant potential of our extracts, so the radical scavenging activity (RSA) of CYS and EUC against different radicals was determined by three easy, rapid and sensitive methods: DPPH, ABTS and FRAP assays. In fact, the combination of several antioxidant procedures should be performed in vitro for a valid assessment of antioxidant activity.

**Figure 1:**
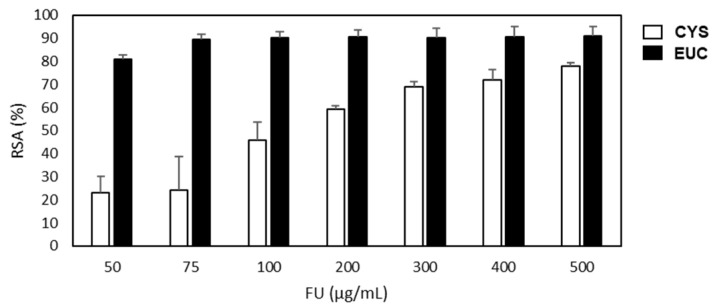
DPPH radical scavenging activity of fucoidan extracted from *C. compressa* (CYS) and *E. globulus* (EUC). RSA = radical scavenging activity (%) = ((absorbance of control−absorbance of sample)/(absorbance of control)) × 100. Values represent mean ± S.D. from triplicate experiments.

As indicated in Figure 1, CYS showed a good ability in scavenging the DPPH free radical (about 20% to 80% inhibition in the concentration range 50–500 µg/mL), but EUC was much stronger as an antioxidant, since 80% RSA was already observed at the concentration of 50 µg/mL. Table 2 reports the IC_50_ values for CYS and EUC with respect to ascorbic acid in the DPPH assay. The complete assay performed to determine EUC IC_50_ is reported in the Supplementary Materials (Figure S4). EUC antioxidant activity was similar to that of ascorbic acid, whereas CYS was about 38 times lower. This is in line with previous reports showing that the scavenging effects of fucoidans extracted from *C. crinita*, *C. compressa* and *C. sedoides* in DPPH assays were less important than those of ascorbic acid used as reference compound [14,27]. Moreover, CYS IC_50_ was very closed to that previously reported by us for *F. hermonis* (IC_50_ = 157.6 ± 3.3 µg/mL) [8]. Results reported in Table 3 confirmed the high antioxidant potential of EUC, almost equal to that of Trolox in ABTS assay, whereas CYS was 100 times weaker. The FRAP values suggest that the significant antioxidant activity of FU extracts could be attributed to different mechanisms, such as complex with transition metal ion catalysts, break of chain initiation, radical scavenging prevention and increase of reductive capacity. Altogether, the results indicated that EUC was stronger than CYS as an antioxidant, and this is in line with the higher TPC value. However, there are several factors that act synergistically to determine the final antioxidant power of FU. The sulfate content and the molecular weight are able to influence the radical scavenging activity of FU [14], and it is conceivable that growing environments as different as the sea and the land can affect the amount and position of sulfate groups and the kind of side chain sugar, thus determining the differences in the antioxidant potential of FU from different sources [28].

### 2.4. Antioxidant and Antisteatotic Activities of CYS and EUC in a Cellular Model of Hepatic Steatosis

Antioxidant agents can be useful as therapeutic compounds against oxidative stress-related diseases. Therefore, we moved to test the antioxidant effects of CYS and EUC in cellular systems. We used an in vitro model of hepatic steatosis consisting in FaO cells overloaded with a mixture of oleate and palmitate (OP, 0.75 mM) to mimic NAFLD [10]. Preliminary results of MTT assay demonstrated that none of the FU extracts tested at different concentrations (5–100 μg/mL) affected cell viability both in the absence and in the presence of OP (data not shown). The intermediate concentration of 50 μg/mL was selected for further experiments.

**Figure 2:**
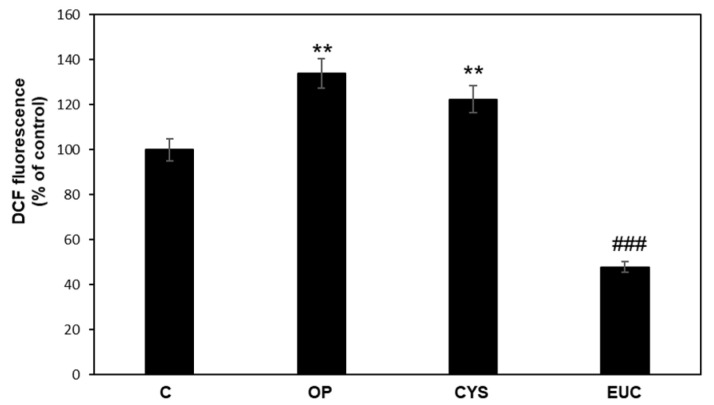
Effects of CYS and EUC on ROS production in steatotic FaO cells. DCF fluorescence was quantified in control (C) and steatotic cells incubated in the absence (OP) or in the presence of 50 μg/mL CYS or EUC for 24 h. Data are expressed as percentage of control. Values are mean ± S.D. from three independent experiments. Significant differences are denoted by symbols: ** *p* ≤ 0.01 vs. control; ### *p* ≤ 0.001 vs. OP.

The antioxidant ability of CYS and EUC was analyzed using 2′,7′-dichlorofluorescein diacetate (DCF-DA) assay for ROS detection in FaO cells (Figure 2). OP treatment resulted in a significant increase in ROS production (+30%, *p* ≤ 0.01 with respect to control), which was not significantly affected by CYS treatment. On the contrary, EUC was able decrease ROS production in OP cells even below control values (*p* ≤ 0.001 vs. OP). This is of interest since oxidative stress can be considered as a starting point of the hepatic damage, acting together with concurrent and/or sequential “multiple hits” that trigger NAFLD progression and worsening toward more severe pathological conditions [29,30].

**Figure 3:**
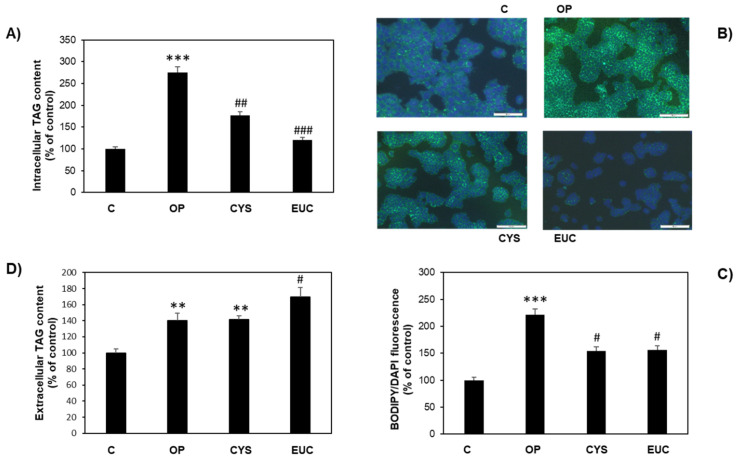
Antisteatotic effect of CYS and EUC on FaO cells. (**A**) Intracellular TAG content in control (**C**) and steatotic cells incubated in the absence (OP) or in the presence of 50 μg/mL CYS or EUC for 24 h. (**B**) Representative images of BODIPY (green)/DAPI (blue) staining of FaO cells showing cytosolic LDs (magnification 10×; bar 50 μm). (**C**) Spectrofluorometric quantification of fluorescence shown in (**B**). (**D**) Extracellular TAG content as measured in the culture medium of the same cells. Data are expressed as percentage of control. Values are mean ± S.D. from three independent experiments. Significant differences are denoted by symbols: ** *p* ≤ 0.01, *** *p* ≤ 0.001 vs. C; # *p* ≤ 0.05, ## *p* ≤ 0.01, ### *p* ≤ 0.001 vs. OP.

Therefore, we investigated whether the antioxidant effect was associated with an antisteatotic action in lipid-overloaded FaO cells. Intracellular triacylglycerol (TAG) content was quantified in both control (C) and steatotic cells in the absence (OP) or in the presence of CYS or EUC using two different methods. Results obtained from intracellular TAG measurement by enzymatic spectrophotometric analysis are reported in Figure 3A and indicated that OP treatment significantly increased TAG content with respect to control (+175%; *p* ≤ 0.001) as previously described [31]. However, incubation with 50 μg/mL of CYS or EUC upon OP treatment decreased TAG content by 35% and 56%, respectively, as compared to OP (*p* ≤ 0.01). The lipid-lowering property of the two FU extracts was confirmed by BODIPY/DAPI staining of FaO cells. Qualitative analysis under fluorescence microscopy allowed the visualization of intracellular lipid droplets (LDs) stained in green with BODIPY 493/503 (Figure 3B). The fluorescence was quantified by spectrofluorimetry to build the bar plot shown in Figure 3C. Moreover, TAG secretion by FaO cells was studied by measuring TAG content in the culture medium of the same cells. Results reported in Figure 3D showed that TAG secretion was significantly increased in OP cells with respect to control (+40%, *p* ≤ 0.01). Subsequent CYS treatment did not affect this value, while EUC treatment enhanced this secretion in a significant way with respect to OP (*p* ≤ 0.05).

**Figure 4:**
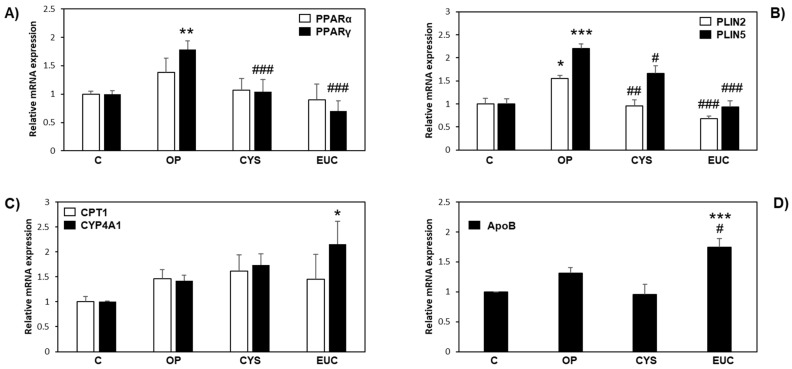
Effects of CYS and EUC on hepatic gene expression. The relative mRNA expression of (**A**) PPARα and PPARγ, (**B**) PLIN2 and PLIN5, (**C)** CPT-1 and CYP4AA1 and (**D**) ApoB was quantified by qPCR in control (**C**) and steatotic FaO cells incubated in the absence (OP) or in the presence of CYS or EUC 50 µg/mL for 24 h. Data are expressed as fold induction with respect to controls. Values are mean ± S.D. from three independent experiments. Significant differences are denoted by symbols: * *p* ≤ 0.05, ** *p* ≤ 0.01, *** *p* ≤ 0.001 vs. C; # *p* ≤ 0.05, ## *p* ≤ 0.01, ### *p* ≤ 0.001 vs. OP.

FaO cells express a wide range of liver-specific mRNAs, which makes this cell line suitable for investigating the effect of FU extracts on key genes regulating hepatic lipid metabolism [32]. Peroxisome proliferator-activated receptors (PPARs) are ligand-activated transcription factors playing a key role in NAFLD. PPARα and PPARγ represent the most abundant PPAR isoforms expressed in FaO cells. Particularly, PPARγ agonists such as thiazolidinediones (rosiglitazone and pioglitazone) represent the more successful therapeutic agents in improving NAFLD histological outcomes in clinical trials [33,34]. PPARγ is a known marker of fatty liver, and its expression is induced in steatotic FaO cells possibly to allow energy storage, as it mediates the activation of lipogenic genes [35,36], as well as the regulation of perilipins (PLINs), a family of LD-associated proteins that confer dynamicity to these organelles, thus taking part in the regulation of lipid trafficking inside and outside the cell [37,38,39,40]. Figure 4A shows that only PPARγ expression was significantly induced by OP treatment (1.5-fold induction, *p* ≤ 0.01) and downregulated to control levels after incubation with both CYS and EUC (*p* ≤ 0.01 as compared to OP). PLIN2 and PLIN5 expression levels, which are regulated by PPARγ, varied accordingly. In fact, steatotic FaO cells showed significantly increased mRNA levels of both PLIN2 and PLIN5, with respect to control (0.5- and 1.2-fold induction, *p* ≤ 0.05 and *p* ≤ 0.001, respectively), while upon treatment with CYS or EUC, the expressions were significantly reduced as compared to OP (Figure 4B).

The antioxidant and antisteatotic effects of CYS and EUC in hepatic cells mimic those exerted in the same model by a FU fraction extracted from *F. hermonis* [8], thus strengthening the possible role of FU as an active constituent against NAFLD [2], even if the mechanisms of action of the different extracts might be different.

As shown in Figure 4C, genes encoding for proteins implicated in lipid catabolism such as carnitine palmitoyl-transferase I (CPT-1) and cytochrome P450 CYP4A1 did not change their expression levels under the different experimental conditions, with the exception of CYP4A1, a cytochrome P450 involved in microsomal ω-oxidation of fatty acids, which was significantly increased by EUC with respect to control (*p* ≤ 0.05). Moreover, Figure 4D demonstrates that EUC was the only treatment able to induce the expression of ApoB mRNA (*p* ≤ 0.001 vs. control, *p* ≤ 0.05 vs. OP). ApoB is the primary apolipoprotein of hepatic VLDL; therefore, the result indicates that EUC can enhance liver lipid secretion, and this is in line with the rise in extracellular TAGs observed under the same experimental condition (Figure 3D). Taken together, these results demonstrate a downregulation by CYS and EUC of energy-storing mechanisms within the liver, with the involvement of changes in LD trafficking. In the case of EUC, it seems that excess fats mobilized from LDs are addressed to microsomal ω-oxidation as well as to TAG secretion through VLDL synthesis, thus resulting in a powerful antisteatotic effect in the liver tissue.

### 2.5. Effects of CYS and EUC on Steatotic Endothelial Cells

**Figure 5:**
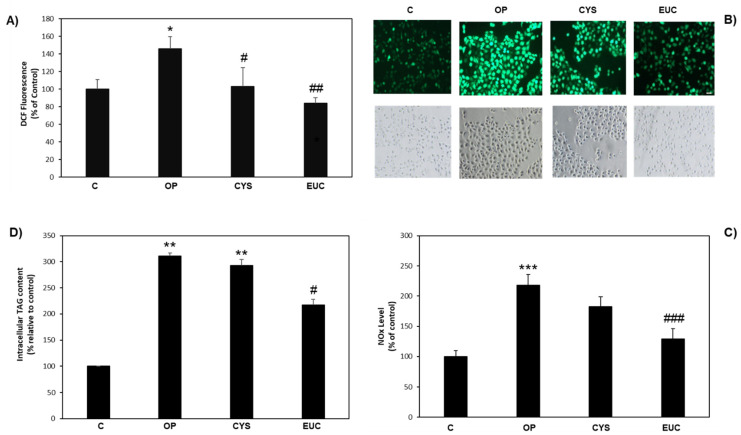
Effects of CYS and EUC in steatotic HECV cells. HECV cells were incubated in the absence (OP) or in the presence of 50 μg/mL CYS or EUC for 24 h. (**A**) Spectrofluorometric quantification of DCF fluorescence. (**B**) Representative images of DCF fluorescence quantified in (**B**) (magnification 10×; bar 50 μm). (**C**) NOx production measurements. (**D)** Intracellular TAG content assay. Data are expressed as percentage relative to control. Values are mean ± S.D. from three independent experiments. Significant differences are denoted by symbols: * *p* ≤ 0.05, ** *p* ≤ 0.01, *** *p* ≤ 0.001 vs. C; # *p* ≤ 0.05, ## *p* ≤ 0.01, ### *p* ≤ 0.001 vs. OP.

OP-treated HECV cells can be considered as a model of atherosclerosis and endothelial damage [11]. Antioxidant capacities of CYS and EUC were confirmed also in HECV cells. OP-treated cells showed a significant increase in ROS production (+46%, *p* ≤ 0.05 with respect to control), as measured by DCF assay. However, both CYS and EUC were able to significantly reduce this rise to control levels (*p* ≤ 0.05 vs. OP), as can be seen in Figure 5A and in the representative images of Figure 5B. Moreover, OP treatment significantly enhanced NO production in HECV cells compared to control (+118%, *p* ≤ 0.001) and this increase was effectively reduced by EUC treatment (−40%, *p* ≤ 0.001 with respect to OP), whereas only a nonsignificant tendency towards a reduction was observed for CYS (Figure 5C). As for intracellular TAG content, the increase induced by OP treatment (+210%, *p* ≤ 0.01 vs. control) was significantly reduced only upon EUC incubation (−30%, *p* ≤ 0.05 vs. OP) (Figure 5D).

Endothelial cells serve as a permeable barrier between blood vessels and tissues to regulate blood flow and allow energy supply, and thus their proper functioning is pivotal, while endothelial dysfunction plays a central role in different metabolic disorders such as obesity, impaired glucose metabolism, hypertension and dyslipidemia [41]. Moreover, endothelial cells are the most abundant nonparenchymal cells in the liver, where they are crucial for the transfer of nutrients, lipids and lipoproteins. Miyao et al. (2015) indicated that endothelial injury might have a “gatekeeper” role in NAFLD worsening toward nonalcoholic steatohepatitis (NASH) [42]. It is well known that in liver sinusoids, NO influences endothelial cell functions related to inflammatory and fibrotic processes [43]. Under pathological conditions such as hypertension, atherosclerosis and angiogenesis-associated disorders, inducible nitric oxidase synthase (iNOS) expression is upregulated, resulting in vascular NO overproduction and transport abnormalities, and increased iNOS levels have been reported in NAFLD patients. As such, our results suggest the potentiality of EUC to counteract endothelial dysfunction in cardiovascular pathologies such as hypertension and atherosclerosis, which are often associated with NAFLD [44,45].

## 3. Materials and Methods

### 3.1. Biological Material and Chemicals

The brown seaweed *C. compressa* (Esper) Gerloff and Nizamuddin was harvested from Tyr Coast, South Lebanon, in the period of April–June 2018. The alga was authenticated by Prof. Hussein Kanaan at the Faculty of Pharmacy, Lebanese University, Hadath, Beirut, Lebanon, as previously described [46]. The blade and stipe of *C. compressa* were washed and cleaned with water and then dried at room temperature and ground for further extraction. The FU fraction from *E. globulus* Labill leaves (EUC) was obtained from the Laboratory of Chemical Synthesis and Extraction of Polysaccharides from Seaweed, Faculty of Pharmacy, Lebanese University, Beirut, Lebanon, where it was previously purified [47]. Unless otherwise indicated, all materials and chemicals were supplied by Sigma-Aldrich Corp. (Milan, Italy). All the reagents were of analytical or cell culture grade (≥95% conforming to ACS specifications).

### 3.2. Extraction and Purification of Fucoidan

Water-soluble polysaccharides were obtained as previously described [9] using slightly modified methods [48,49]. Briefly, 100 g of *C. compressa* was extracted twice with 250 mL absolute ethanol for 3 h at 40 °C to remove low-molecular-weight compounds such as pigments, phenols and proteins. The dried residues were extracted twice with aqueous HCl solution (pH = 2) at 60 °C for 3 h and then centrifuged at 1600× *g* for 20 min to obtain supernatant containing the FLM complex (fucoidan, laminarin, mannuronan). The supernatant was neutralized with 3% NaHCO_3_ and evaporated in a Rotavac Vario Power Unit (Heidolph Instruments, Schwabach, Germany) to a final volume of 200 mL, which was subjected to 24 h dialysis (Spectra/Por Dialysis Tubing, MWCO 12,000–14,000) and subsequent lyophilization to obtain FLM powder (Alpha 1-4 LD plus lyophilizer, CHRIST, Osterode am Harz, Germany). Fucoidan purification was achieved by adding 50 mL of aqueous HCl solution (pH = 2), followed by centrifugation at 1620× *g* for 20 min. The pellet was discarded, and the supernatant was lyophilized to obtain dry powdered fucoidan (CYS), which was weighed to calculate yield.

### 3.3. Chemical Characterization

Sulfate and protein contents were determined by the turbidimetric assay described by Jackson and McCandless [50]. Absorbance was read at 500 nm with a Varian Cary 50 UV-VIS spectrophotometer (Agilent, Milan, Italy).

Sugar content was quantified using the phenol–sulfuric acid method developed by DuBois et al. [51]. Briefly, 2 mL aliquots of the extracts were incubated with 0.5 mL of phenol (3% *v*/*v*) and 5 mL of H_2_SO_4_ for 15 min at RT followed by absorbance recording at 520 nm. Fucose, glucose, galactose and mannose were used as standards.

Total phenolic content (TPC) was determined using the Folin–Ciocalteu method [52]. Briefly, 100 μL aliquots of each extract (1 mg/mL) were incubated with 0.5 mL of 10% (*w*/*v*) Folin–Ciocalteu reagent. After 5 min, 1.5 mL of Na_2_CO_3_ (2% *w*/*v*) was added and incubated in the dark at room temperature for 30 min. The absorbance was measured at 760 nm using a Hitachi U-2900 UV-Vis spectrophotometer (Hitachi High-Technologies, Tokyo, Japan). Results were expressed in μg of gallic acid equivalents (GAE)/mg dry extract weight.

Fourier transform infrared spectroscopy (FTIR) was conducted using a PerkinElmer FTIR spectrometer Spectrum Two UAT (PerkinElmer Italia SpA, Milan, Italy). Data were collected in the range of 4000–400 cm^−1^. Proton (^1^H NMR) and carbon (^13^C NMR) nuclear magnetic resonance spectroscopy were conducted by analyzing NMR spectra using a Bruker Ascend 500 AVANCE III HD spectrometer (Bruker Italia SRL, Milan, Italy). The water-soluble polysaccharide was dissolved in 99% deuterium oxide (D_2_O), and the spectra were recorded at room temperature (^1^H NMR: frequency 500 MHz, acquisition time 3.27 s; ^13^C NMR: frequency 125 MHz, acquisition time 1.1 s). Data are reported in Supplementary Materials.

### 3.4. Radical Scavenging Capacity

#### 3.4.1. 2,2-Diphenyl-1-Picrylhydrazyl (DPPH) Radical Assay

The capacity of CYS and EUC to scavenge the free radical 2,2-diphenyl-1-picrylhydrazyl (DPPH) was determined basically according to the method described by Haddad et al. [9]. One-milliliter aliquots of CYS or EUC at different concentrations (2.5, 5, 10, 25, 50, 75, 100, 200, 300, 400 and 500 μg/mL) were prepared and mixed with 1 mL of DPPH solution (0.05 g/L in methanol). After a 30 min incubation in darkness, the DPPH radical reduction was evaluated by reading the absorbance at 517 nm using a Gene Quant 1300 UV-Vis spectrophotometer (Biochrom Ltd, Cambridge, UK). Ascorbic acid was used as reference standard. The results were calculated as follows: DPPH scavenging activity (%) = ((absorbance of control − absorbance of sample)/(absorbance of control)) × 100. The IC_50_ value, defined as the concentration of compound required to cause a 50% decrease in initial DPPH concentration, was estimated by the plot of percentage of inhibition vs. concentration of extract, using a nonlinear regression algorithm (logarithmic) and GraphPad software (GraphPad Software, Inc., San Diego, CA, USA).

#### 3.4.2. 2,2-Azinobis-3-Ethylbenzothiazoline-6-Sulfonic Acid (ABTS) Assay

The ABTS assay was performed following the procedure described previously [53], applied to a 96-well microplate. The ABTS^·+^ stock solution was prepared according to [54] to obtain a final solution with the absorbance of about 1 at 734 nm. FU extracts at different concentrations (6.25, 12.5, 25, 50, 100, 200 and 400 μg/mL) were incubated with ABTS^·+^ for 10 min at room temperature in the dark, and then the absorbance was read at 734 nm using a microplate reader (FLUOstar Optima, BMG Labtech microplate reader, BioTek Instruments, Winooski, VT, USA). Trolox was used as reference, and results were expressed as µmol Trolox Equivalents (TE)/g of extract (µmol TE/g). The IC_50_ value, defined as the concentration of compound required to cause a 50% reduction in the assay, was estimated by the plot of percentage of inhibition vs. concentration of extract, using a nonlinear regression algorithm (logarithmic) and GraphPad software.

#### 3.4.3. Ferric Reducing Antioxidant Power (FRAP) Assay

FRAP assay was performed as previously reported [53] by using a 96-well microplate to monitor the reduction of Fe^3+^ tripyridyl triazine (TPTZ) to blue-colored Fe^2+^-TPTZ. Fresh working solution was prepared by mixing 10 volumes of acetate buffer (300 mM, pH 3.6), 1 volume of TPTZ (10 mM in 40 mM HCl) and 1 volume of FeCl_3_·6 H_2_O (20 mM). CYS or EUC (concentrations as for ABTS assay) were incubated with FRAP solution for 10 min, and the absorbance was read at 593 nm with Trolox as standard as described above.

### 3.5. Cell Culture and Treatments

FaO rat hepatoma cell line (European Collection of Authenticated Cell Cultures, ECACC, Salisbury, Wiltshire, UK) was grown in Coon’s modified Ham’s F-12 medium supplemented with L-glutamine and 10% fetal bovine serum (FBS).

Human endothelial cord vein (HECV) cells (Cell Bank and Culture-GMP-IST-Genoa, Italy) were grown in Dulbecco’s modified Eagle’s medium (DMEM) supplemented with L-glutamine and 10% FBS.

Cells were incubated in a humidified atmosphere with 5% CO_2_ at 37 °C. For treatments, cells were grown until 80% confluence and incubated overnight in serum-free medium with 0.25% bovine serum albumin. To induce intracellular lipid accumulation, cells were treated for 3 h with a mixture of oleate and palmitate (OP) at a final concentration of 0.75 mM (2:1 molar ratio) [55]. Thereafter, cells were incubated for 24 h either in control medium (referred to as OP steatotic cells) or in the presence of FU extracts at different concentrations, starting from an aqueous stock solution (1 mg/mL) diluted with culture medium. Untreated cells were referred to as controls.

### 3.6. ROS Production

ROS production in FaO and HECV cells was quantified following the oxidation of the cell-permeant 2′-7′-dichlorofluorescein diacetate (DCF-DA, Fluka GmbH, Neu-Ulm, Germany) to 2′-7′-dichlorofluorescein (DCF). A stock solution of DCF-DA (10 mM in DMSO) was prepared and stored at −20 °C in the dark. At the end of treatments, cells were scraped and centrifuged (600× *g* for 10 min at 4 °C). After washing with PBS, cells were loaded with 10 μM DCF-DA in PBS and incubated for 30 min at 37 °C in the dark. Then, cells were centrifuged and resuspended in PBS, and the fluorescence was measured fluorometrically (λex = 495 nm; λem = 525 nm) in an LS50B fluorimeter (PerkinElmer, MA, USA) at 25 °C using a water-thermostated cuvette holder. Results were normalized for protein content [56]. The intracellular ROS production was also visualized in situ by fluorescence microscopy of DCF-stained cells. Images were acquired with a Leica DMRB light microscope equipped with a Leica CCD camera DFC420C (Leica, Wetzlar, Germany).

### 3.7. Quantification of Triglycerides (TAGs)

Intracellular TAG content was measured using the “Triglycerides liquid” kit (Sentinel, Milan, Italy), as previously described [57,58]. The absorbance was recorded at 546 nm using a Varian Cary50 spectrophotometer (Agilent, Milan, Italy). For measurement of extracellular TAG content, the culture media were processed according to the same method. Values were normalized to protein content, and data are expressed as percent TAG content relative to controls [59]. For intracellular lipid staining, cells grown on coverslips were rinsed with PBS and fixed with 4% paraformaldehyde for 20 min at room temperature. Neutral lipids were stained by incubation with 1 μg/mL BODIPY 493/503 (Molecular Probes, Life Technologies, Monza, Italy) in PBS for 30 min [60]. After washing, nuclei were stained with 4′,6-diamidino-2-phenylindole (DAPI), 5 μg/mL (ProLong Gold medium with DAPI; Invitrogen, MA, USA). Mounted slides were examined at 10X magnification by Olympus IX53 light microscope (Olympus, Milano, Italy), equipped with the standard epifluorescence filter setup. Representative images were captured with a CCD UC30 camera (Olympus) and digital image acquisition software (CellSens Entry, Olympus).

### 3.8. Nitric Oxide (NO) Production

NO production by HECV cells was measured indirectly by spectrophotometric quantification of the end products (nitrites and nitrates, collectively referred to as NOx) at 540 nm, using the Griess reaction [61]. NOx accumulation in cell culture media was calculated against a standard curve of sodium nitrite (NaNO_2_) and normalized by protein content (μmol NaNO_2_/mg sample protein) [56].

### 3.9. RNA Extraction and Quantitative Real-Time PCR

Total RNA was extracted by using TRI Reagent (Sigma-Aldrich, Oakville, ON, Canada, and St. Louis, MO, USA) according to the manufacturer’s instructions (https://www.sigmaaldrich.com/IT/it/technical-documents/protocol/protein-biology/protein-lysis-and-extraction/tri-reagent, accessed on 18 July 2021). One microgram of cDNA was synthesized using RevertAid H-Minus M-MuLV Reverse Transcriptase (Fermentas, Hannover, MD, USA) as previously explained [62]. Real-time quantitative PCR (qPCR) reactions were performed in triplicate in a final volume of 25 μL using 1 × SybrGreen SuperMix and Chromo4TM System apparatus (Biorad, Monza, Italy) as previously described [19]. Primer pairs for the genes under analysis (Table S1, Supplementary Materials) were designed ad hoc and synthesized by TibMolBiol custom oligonucleotide synthesis service (Genova, Italy). Amplification conditions were as follows: 3 min at 95 °C, followed by 5 s at 95 °C and 1 min at 60 °C or 64 °C for 40 cycles. A melting curve of qPCR products (65–94 °C) was also obtained to ensure the absence of artifacts. The relative quantity of target mRNA was calculated by the comparative Cq method using glyceraldehyde 3-phosphate dehydrogenase (GAPDH) as housekeeping gene and expressed as fold change with respect to controls [63].

### 3.10. Statistical Analysis

Data are means ± S.D. of at least three independent experiments. Statistical analysis was performed using ANOVA with Tukey’s post-test (GraphPad Software, Inc., San Diego, CA, USA).

## 4. Conclusions

Two fucoidan fractions from both marine (CYS) and terrestrial (EUC) sources displayed radical scavenging activities, antioxidant properties and antisteatotic actions. EUC exerted stronger effects than CYS, thus suggesting that terrestrial fucoidans should be considered as new potential active compounds against NAFLD and related diseases.

## Figures and Tables

**Table 1:** Chemical composition (mg/g) of fucoidan extracted from *C. compressa* (CYS) and *E. globulus* (EUC).

	Fucose	Glucose		Galactose			Mannose	Sulfate	Proteins	TPC
**CYS**	222.6 ± 2.7	211.6 ± 5.3		19.8 ± 3.1			9.8 ± 2.9	12.6 ± 6.1	4.2 ± 6.3	19.7 ± 2.2
	**Monosaccharide Composition (Molar ratio)**									
	100		86.7		8.1	4.0				
**EUC**	335.1 ± 3.4		321.7± 4.7		28.9 ± 2.6	14.0 ± 3.7		21.8 ± 6.2	5.9 ± 5.5	139.6 ± 0.9
	**Monosaccharide Composition (Molar ratio)**									
	100		87.6		7.9	3.8				

TPC = total phenolic content, with results expressed as mg GAE/g. Monosaccharide composition is reported as molar ratio with respect to fucose set at 100. All values represent mean ± S.D. from triplicate experiments.

**Table 2:** Antioxidant activity of fucoidan extracted from *C. compressa* (CYS) and *E. globulus* (EUC).

	DPPH IC_50_ (µg/mL)
CYS	152.7 ± 3.2
EUC	4.5 ± 3.3
Ascorbic acid	4.1 ± 2.1

IC_50_ = the concentration of compound that affords a 50% reduction in the DPPH assay. Values represent mean ± S.D. from triplicate experiments.

**Table 3:** Antioxidant activities of CYS and EUC measured by ABTS and FRAP assays.

	ABTS		FRAP
	TEAC (μmol TE/g)	IC_50_ (μg/mL)	TEAC (μmol TE/g)
CYS	39.0 ± 3.7	478.6 ± 39.2	77.2 ± 9.1
EUC	1444.1 ± 11.5	12.9 ± 0.8	638.9 ± 17.1
Trolox		4.5 ± 1.5	

TEAC = Trolox equivalent (TE) antioxidant concentration. IC_50_ = the concentration of compound that affords a 50% reduction in the assay. Values represent mean ± S.D. from triplicate experiments.

## Data Availability

The data presented in this study are available in Supplementary Materials.

## Supplementary Materials

The following are available online. Figure S1: FTIR spectrum of fucoidan isolated from *C. compressa*. Figure S2: ^1^H NMR spectrum of fucoidan isolated from *C. compressa*, Figure S3: ^13^C NMR spectrum of fucoidan isolated from *C. compressa*, Table S1: Primer pairs used for RT-qPCR analysis.

## Sample Availability

Samples of the fucoidan extracts CYS and EUC are available from the authors.
