# Supplementary material for: Antioxidant and Antisteatotic Activities of Fucoidan Fractions from Marine and Terrestrial Sources
Source: Molecules. 2021 Jul 24;26(15):4467. doi: 10.3390/molecules26154467 (PMC8347863; doi:10.3390/molecules26154467)
Supplement: Supplementary file 1 [file molecules-26-04467-s001.zip › molecules-1298684-supplementary.pdf]

Supplementary Materials

Antioxidant and antisteatotic activities of fucoidan fractions from marine and terrestrial sources.

Zeinab El Rashed [1,2], Giulio Lupidi [3], Elena Grasselli [1], Laura Canesi [1], Hala Khalifeh [2] and Ilaria Demori [1,*]

[1] Department of Earth, Environmental and Life Sciences (DISTAV), University of Genoa, 16132 Genoa, Italy; Zeinab.ALRashed94@hotmail.com (Z.E.R.), elena.grasselli@unige.it (E.G.), laura.canesi@unige.it (L.C.), idemori@unige.it (I.D.)
[2] Rammal Rammal Laboratory (ATAC group), Faculty of Sciences I, Lebanese University, 1003 Beirut, Lebanon.; Zeinab.ALRashed94@hotmail.com (Z.E.R.), hala-khalifeh@hotmail.com (H.K.)
[3] School of Pharmacy, University of Camerino, 62032 Camerino, Italy; giulio.lupidi@unicam.it (G.L.)
*Correspondence: idemori@unige.it; Tel.: +390103538246

FTIR spectroscopic analysis

Infrared spectroscopy (FTIR) of CYS was recorded on a Perkin-Elmer FTIR spectrometer Spectrum Two UAT. Data were collected in the range of 4000-400 cm^{-1}.

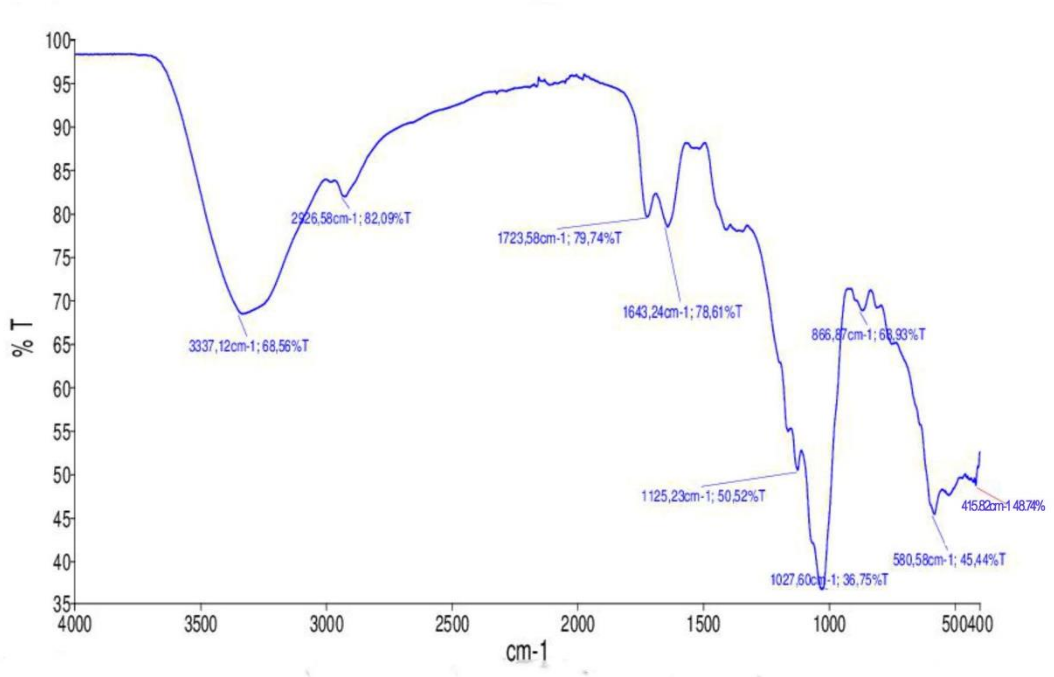

Figure S1. FTIR spectrum of fucoidan isolated from *C. compressa*. %T: % Transmittance.

Nuclear magnetic resonance spectroscopy

Proton (^1H NMR) and carbon (^{13}C NMR) nuclear magnetic resonance spectroscopy were determined by analyzing NMR spectra using a Bruker Ascend 500 AVANCE III HD spectrometer. The water-soluble

polysaccharide was dissolved in 99% deuterium oxide (D_2O), and the spectra were recorded at room temperature (^{1}H NMR: frequency 500 MHz, acquisition time 3.27 sec; ^{13}C NMR: frequency 125 MHz, acquisition time 1.1 sec).

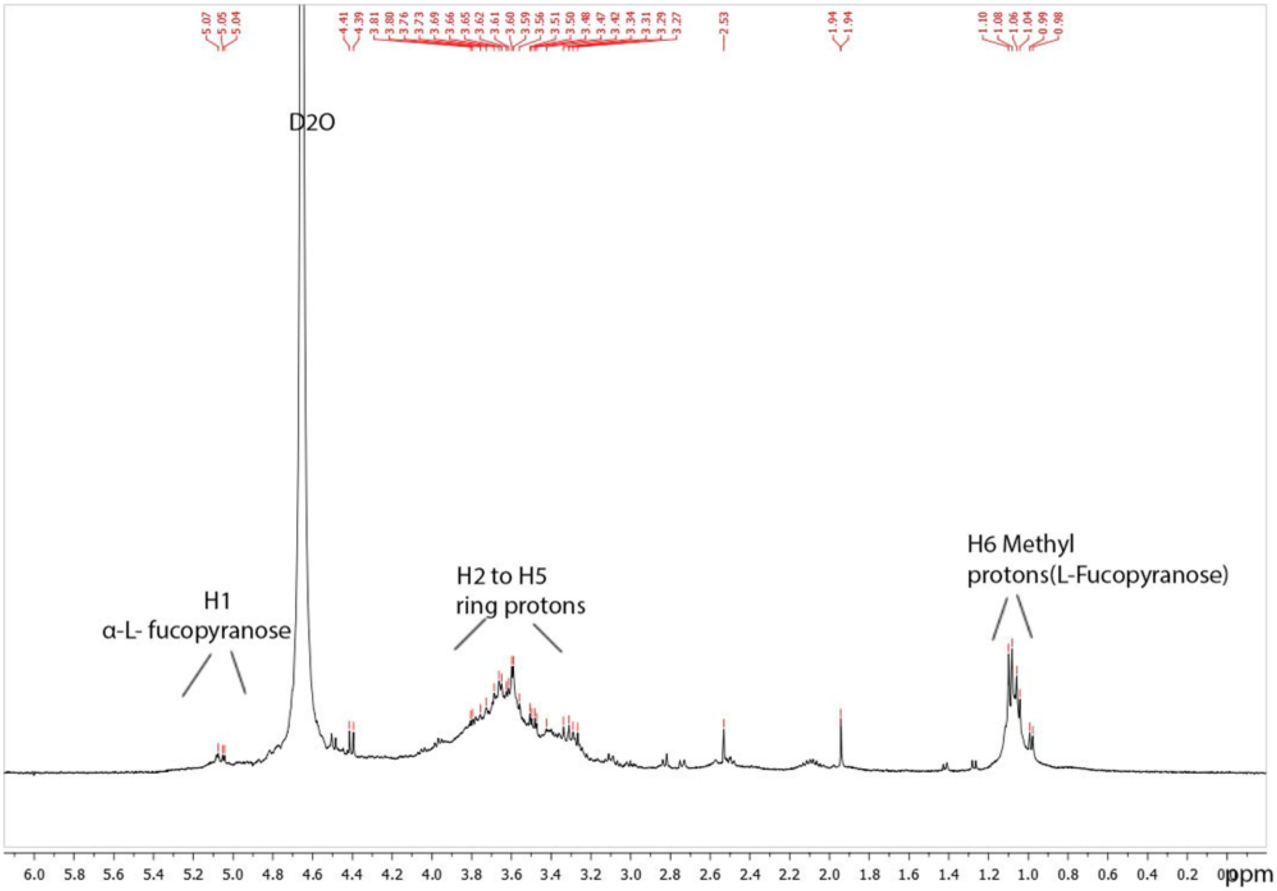

Figure S2: ^{1}H NMR spectrum of fucoidan isolated from *C. compressa.*

The ^{1}H NMR spectrum of CYS reported in Figure S2 gave relatively minor informations. Similarly to that previously reported for *F. hermonis* [1] the spectrum showed broad signals of methyl peak at 1.0–1.1 ppm, corresponding to H6 methyl protons of L-fucopyranosyl units. A single minor H1 signal of lower intensity resonated at 5.04 - 5.07, and that could be consistent with the presence of α-L-fucopyranose [2]. The spectrum also contained resonance characteristics of fuicodan derivatives, with spread signals from ring protons (H2 to H5) at 3.27 to 3.90 ppm, indicating the presence of different types of fucosal sulfate groups with changes in glycosidic bound position and monosaccharide types [2]. Minor signals of lower intensity, that appear between 4.39 and 4.41 ppm might correspond to ring protons of D-glucose and D-galactose [3].

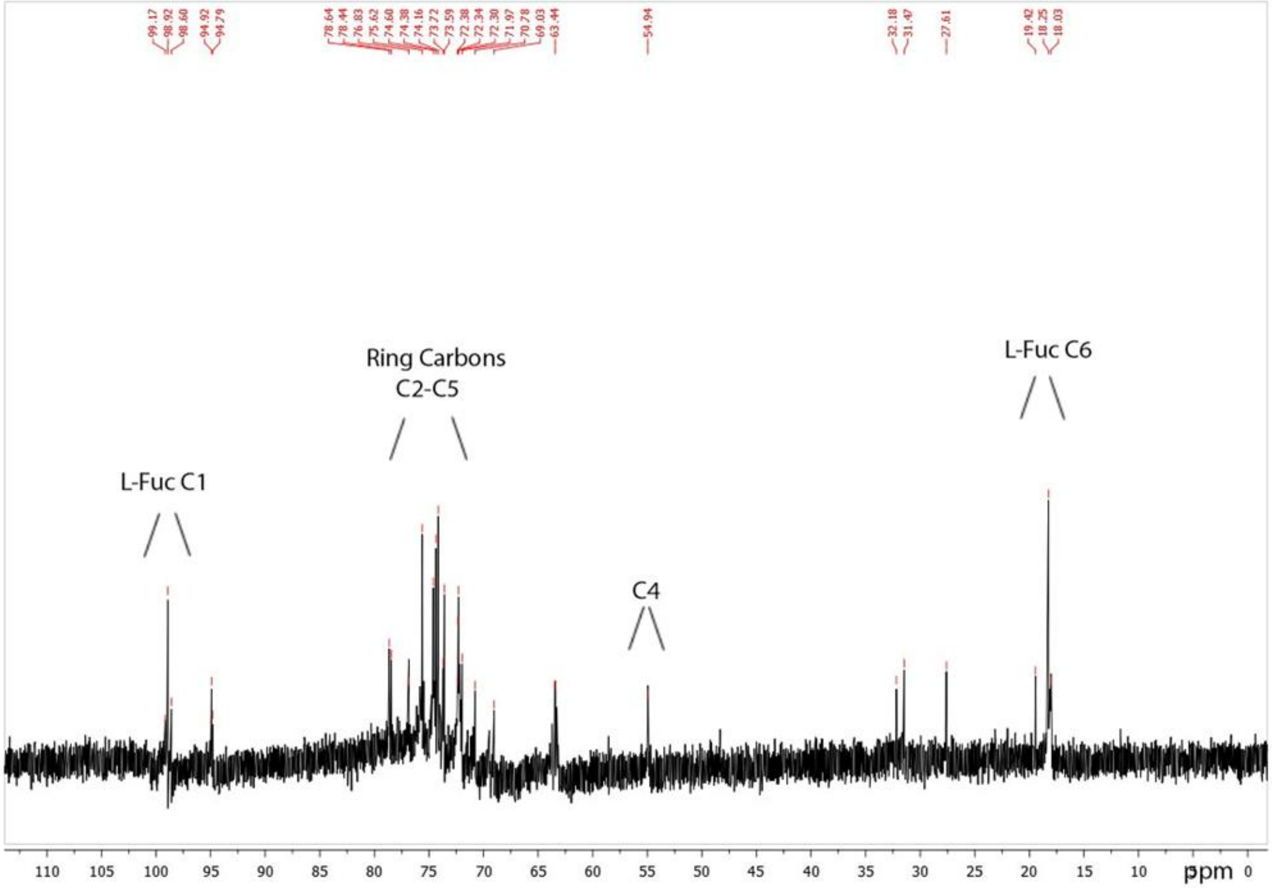

Figure S3. ^{13}C NMR spectrum of fucoidan isolated from *C. compressa*.

The ^{13}C NMR spectrum of CYS reported in Figure S3 was more complex. Intense peaks between 92.3-97 ppm and 107 ppm (C1) can be assigned to the anomeric region of sulfated L-fucan. A broad intense signal at a higher field region between 15.5 and 16.8 ppm represented α-fucopyranosides (C6) as reported also for other fuicodan derivatives [4,5]. The spread signals of pyranoid ring carbons (C2-C5) obtained in the region between 60.7 and 76.0 ppm represent the complex structural pattern of monomeric composition of fucoidan as also its sulfated or glycosylated patterns. The signal at 52.2 ppm is tentatively attributed to C4 [1,6-8].

Altogether, FTIR and NMR spectra of CYS indicated the presence of main functional groups that characterize FU, such as L-fucopyranosyl units and sulfate groups. However, further spectrophotometric techniques and chemical characterization studies are needed to better investigate the detailed structure of CYS regarding sulfation, O-acetylation, linkage, and branching points of the backbone chain.

Regarding EUC, FTIR and NMR spectra have been previously published [9]. When comparing our data with those already obtained for EUC, minor differences in peaks can be detected, that could be related to the diversity of the growth environment.

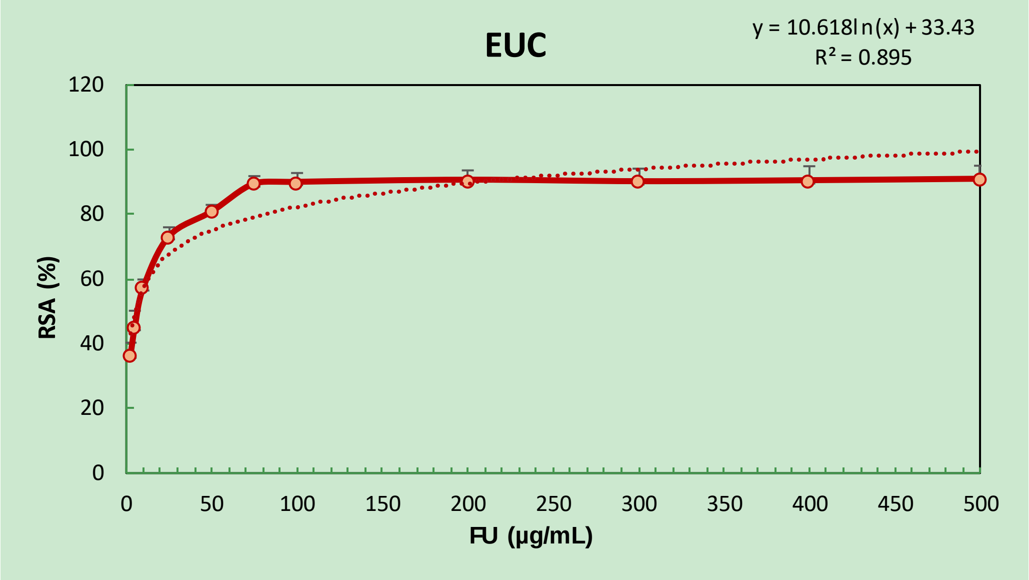

Figure S4. Radical scavenging activity of fucoidan extracted from *E. globulus* (EUC). RSA = radical scavenging activity (%) = [(absorbance of control − absorbance of sample)/(absorbance of control)] × 100. Values represent mean ± S.D. from triplicate experiments. Results refer to DPPH assay.

Quantitative real-time PCR

Table S1. Primer pairs used for RT-qPCR analysis

PRIMER NAME	Primer sequence (5′→3′)	Annealing T (°C)	Accession ID
GAPDH Fwd	GACCCCTTCATTGACCTCAAC	60	DQ403053
GAPDH Rev	CGCTCCTGGAAGATGGTGATGGG		
PPARα Fwd	CCCCACTTGAAGCAGATGACC	60	NM_013196
PPARα Rev	CCCTAAGTACTGGTAGTCCGC		
PPARγ Fwd	CGGAGTCCTCCCAGCTGTTCGCC	60	Y12882
PPARγ Rev	GGCTCATATCTGTCTCCGTCTTC		
PLIN2 Fwd	CCGAGCGTGGTGACGAGGG	60	AAH85861
PLIN2 Rev	GAGGTCACGGTCCTCACTCCC		
PLIN5 Fwd	GGATGTCCGGTGATCAGAC	60	XM_576698
PLIN5 Rev	GTGCACGTGGCCCTGACCAG		
CPT1 Fwd	CCGCTCATGGTCAACAGCA	60	NM_031559
CPT1 Rev	CAGCAGTATGGCGTGGATGG		
CYP4A1 Fwd	CCGGCTCATAACCCATCAACT	64	NM_175837
CYP4A1 Rev	AACGTGGAAGGTGCTTCACTGC		
ApoB Fwd	CGTGGGCTCCAGCATTCTA	60	NM_019287.2
ApoB Rev	TCACCAGTCATTTCTGCCTTTG		

References

1. El Rashed, Z.; Lupidi, G.; Kanaan, H.; Grasselli, E.; Canesi, L.; Khalifeh, H.; Demori, I. Antioxidant and Antisteatotic Activities of a New Fucoidan Extracted from Ferula hermonis Roots Harvested on Lebanese Mountains. *Molecules* **2021**, *26*, doi:10.3390/molecules26041161.
2. Saboural, P.; Chaubet, F.; Rouzet, F.; Al-Shoukr, F.; Azzouna, R.; Bouchemal, N.; Picton, L.; Louedec, L.; Maire, M.; Rolland, L., et al. Purification of a Low Molecular Weight Fucoidan for SPECT Molecular Imaging of Myocardial Infarction. *Marine Drugs* **2014**, *12*, 4851-4867, doi:10.3390/md12094851.
3. Patankar, M.S.; Oehninger, S.; Barnett, T.; Williams, R.L.; Clark, G.F. A revised structure for fucoidan may explain some of its biological activities. *J Biol Chem* **1993**, *268*, 21770-21776, doi:10.1016/S0021-9258(20)80609-7.
4. Alwarsamy, M.; Gooneratne, R.; Ravichandran, R. Effect of fucoidan from Turbinaria conoides on human lung adenocarcinoma epithelial (A549) cells. *Carbohydrate Polymers* **2016**, *152*, 207-213, doi:10.1016/j.carbpol.2016.06.112.
5. Chandia, N. P.; Matsuhiro, B. Characterization of a fucoidan from Lessonia vadosa (Phaeophyta) and its anticoagulant and elicitor properties. *Int J Biol Macromol* **2008**, *42*, 235–240, doi:10.1016/j.ijbiomac.2007.10.023.
6. Haddad, M.; Zein, S.; Shahrour, H.; Hamadeh, K.; Karaki, N.; Kanaan, H. Antioxidant activity of water-soluble polysaccharide extracted from Eucalyptus cultivated in Lebanon. *Asian Pacific Journal of Tropical Biomedicine* **2017**, *7*, 157-160, doi:10.1016/j.apjtb.2016.11.024.
7. Cho, M.L.; Lee, B.-Y.; You, S.G. Relationship between Oversulfation and Conformation of Low and High Molecular Weight Fucoidans and Evaluation of Their in Vitro Anticancer Activity. *Molecules* **2010**, *16*, 291-297, doi:10.3390/molecules16010291.
8. Somasundaram, S.N.; Shanmugam, S.; Subramanian, B.; Jaganathan, R. Cytotoxic effect of fucoidan extracted from Sargassum cinereum on colon cancer cell line HCT-15. *International Journal of Biological Macromolecules* **2016**, *91*, 1215-1223, doi:10.1016/j.ijbiomac.2016.06.084.
9. Zein, S.; Haddad, M.; Krivoruchko, E.; Sobolev, A.P.; Azar, S.; Kanaan, H. A New Molecule of Water-Soluble Polysaccharide Isolated from Eucalyptus Growing in Lebanon. *EJPMR* **2018**, *15*, 7.
